# Supplementary material for: Grading of Intraventricular Hemorrhage and Neurodevelopment in Preterm <29 Weeks’ GA in Canada
Source: Children (Basel). 2022 Dec 12;9(12):1948. doi: 10.3390/children9121948 (PMC9777052; doi:10.3390/children9121948)
Supplement: Supplementary file 1 [file children-09-01948-s001.zip › children-2027485-supplementary.pdf]

children MDPI

Supplementary

Table S1. Comparison of outcomes at 18-24 months corrected age among IVH groups among infants born <26 weeks gestation.

Outcome	Level	Unadjusted Odds Ratio (95% CI)	Adjusted Odds Ratio (95% CI)[1]	Adjusted Odds Ratio (95% CI)[2]
NDI	Grade I-II vs. No IVH	0.94 (0.66, 1.34)	0.86 (0.59, 1.26)	0.86 (0.59, 1.26)
	Grade III vs. No IVH	3.15 (1.40, 7.12)	2.76 (1.15, 6.66)	2.84 (1.17, 6.86)
	Grade IV vs. No IVH	2.29 (1.34, 3.93)	2.01 (1.13, 3.58)	2.01 (1.13, 3.59)
sNDI	Grade I-II vs. No IVH	1.30 (0.84, 2.02)	1.26 (0.79, 2.02)	1.24 (0.77, 2.00)
	Grade III vs. No IVH	2.45 (1.17, 5.10)	2.35 (1.06, 5.22)	2.59 (1.15, 5.87)
	Grade IV vs. No IVH	3.91 (2.31, 6.60)	3.53 (1.97, 6.33)	3.40 (1.87, 6.17)

Abbreviations: CI–confidence interval; NDI–neurodevelopmental impairment; sNDI–significant neurodevelopmental impairment.

[1]Adjusted for gestational age, antenatal corticosteroids, sex, small for gestational age, outborn, cesarean delivery, maternal hypertension, Score for Neonatal Acute Physiology-II, early onset sepsis and center.

[2]Adjusted for gestational age, antenatal corticosteroids, sex, small for gestational age, outborn, cesarean delivery, maternal hypertension, Score for Neonatal Acute Physiology-II, sepsis, necrotizing enterocolitis, bronchopulmonary dysplasia, retinopathy of prematurity, and center.

"No IVH" as the reference group.

Table S2. Comparison of outcomes at 18-24 months corrected age among IVH groups among infants born between 26-28$^{6/7}$ weeks gestation.

Outcome	Level	Unadjusted Odds Ratio (95% CI)	Adjusted Odds Ratio (95% CI)[1]	Adjusted Odds Ratio (95% CI)[2]
NDI	Grade I-II vs. No IVH	1.15 (0.91, 1.46)	1.12 (0.86, 1.46)	1.11 (0.85, 1.45)
	Grade III vs. No IVH	2.45 (1.43, 4.19)	2.37 (1.26, 4.45)	2.34 (1.24, 4.40)
	Grade IV vs. No IVH	3.57 (2.27, 5.60)	3.11 (1.92, 5.05)	3.01 (1.84, 4.91)
sNDI	Grade I-II vs. No IVH	1.66 (1.18, 2.35)	1.60 (1.08, 2.38)	1.60 (1.08, 2.38)
	Grade III vs. No IVH	5.56 (3.17, 9.77)	4.97 (2.48, 9.97)	4.95 (2.45, 9.98)
	Grade IV vs. No IVH	5.71 (3.61, 9.03)	5.93 (3.50, 10.0)	5.60 (3.29, 9.52)

Abbreviations: CI–confidence interval; NDI–neurodevelopmental impairment; sNDI–significant neurodevelopmental impairment.

[1]Adjusted for gestational age, antenatal corticosteroids, sex, small for gestational age, outborn, cesarean delivery, maternal hypertension, Score for Neonatal Acute Physiology-II, early onset sepsis and center.

[2]Adjusted for gestational age, antenatal corticosteroids, sex, small for gestational age, outborn, cesarean delivery, maternal hypertension, Score for Neonatal Acute Physiology-II, sepsis, necrotizing enterocolitis, bronchopulmonary dysplasia, retinopathy of prematurity, and center.

"No IVH" as the reference group.
